# Human factors methods in the design of digital decision support systems for population health: a scoping review

**DOI:** 10.1186/s12889-024-19968-8

**Published:** 2024-09-10

**Authors:** Holland M. Vasquez, Emilie Pianarosa, Renee Sirbu, Lori M. Diemert, Heather Cunningham, Vinyas Harish, Birsen Donmez, Laura C. Rosella

**Affiliations:** 1 Department of Mechanical and Industrial Engineering, University of Toronto, Toronto, Ontario Canada; 2 Dalla Lana School of Public Health, University of Toronto, Toronto, Ontario Canada; 3 Gerstein Science Information Centre, University of Toronto, Toronto, Ontario Canada; 4 Temerty Faculty of Medicine, University of Toronto, Toronto, Ontario Canada; 5 Institute for Better Health, Trillium Health Partners, Mississauga, Ontario Canada

**Keywords:** Human factors engineering, Public health, Decision-making tool, User-centered design, Literature review

## Abstract

**Background:**

While Human Factors (HF) methods have been applied to the design of decision support systems (DSS) to aid clinical decision-making, the role of HF to improve decision-support for population health outcomes is less understood. We sought to comprehensively understand how HF methods have been used in designing digital population health DSS.

**Materials and methods:**

We searched English documents published in health sciences and engineering databases (Medline, Embase, PsychINFO, Scopus, Comendex, Inspec, IEEE Xplore) between January 1990 and September 2023 describing the development, validation or application of HF principles to decision support tools in population health.

**Results:**

We identified 21,581 unique records and included 153 studies for data extraction and synthesis. We included research articles that had a target end-user in population health and that used HF. HF methods were applied throughout the design lifecycle. Users were engaged early in the design lifecycle in the needs assessment and requirements gathering phase and design and prototyping phase with qualitative methods such as interviews. In later stages in the lifecycle, during user testing and evaluation, and post deployment evaluation, quantitative methods were more frequently used. However, only three studies used an experimental framework or conducted A/B testing.

**Conclusions:**

While HF have been applied in a variety of contexts in the design of data-driven DSSs for population health, few have used Human Factors to its full potential. We offer recommendations for how HF can be leveraged throughout the design lifecycle. Most crucially, system designers should engage with users early on and throughout the design process. Our findings can support stakeholders to further empower public health systems.

**Supplementary Information:**

The online version contains supplementary material available at 10.1186/s12889-024-19968-8.

## Background

Interactive decision aid systems, such as dashboards, are vital digital interfaces that support decision-makers across diverse sectors like healthcare, energy, and finance [1]. In these dynamic and often unpredictable settings, professionals must make swift and accurate decisions under pressure, where the cost of an error can be substantial. Given that human cognitive and perceptual constraints can lead to decision-making errors, these systems aim to minimize errors and enhance user decision-making.

Human Factors (HF) Engineering, along with its subdiscipline of Human Computer Interaction (HCI), represents a field poised at the intersection of human behavior and system design [2]. It is predicated on the principle of tailoring systems to match user capabilities and characteristics, thereby minimizing the mismatch between humans and the tools they use. This alignment aims to reduce cognitive and physical strain, facilitating improved performance and satisfaction. The methodologies encompass understanding user-system interactions, crafting solutions responsive to user needs, and evaluating these solutions against criteria such as decision-making accuracy, task efficiency, mental workload, and user satisfaction [2].

The emergence of HCI as a distinct field in the late 20th century represents an evolution of the HF tradition, with a specific focus on the interfaces between humans and computers [3]. While the field of HF broadly addresses the design of systems with human users, HCI hones in on the complexities of human interactions with computer systems. HCI researchers examine how individuals interact with computers, striving to make these interactions more intuitive, efficient, and pleasant. This includes studying user behavior, developing new interaction techniques, designing user interfaces, and evaluating user experiences. The relationship between HCI and HF is synergistic; while HF provides the overarching principles of user-centered design and system optimization, HCI applies these principles specifically to the design and evaluation of software systems. Throughout the manuscript we refer to HF in a broad sense, thereby encompassing HCI.

The system design process for software systems begins with a needs assessment and design requirements phase, where user, task, environment, and stakeholder analyses are conducted to define functional, non-functional, user, and regulatory requirements. This is followed by design and prototyping, involving conceptual and detailed design, as well as creating low-fidelity and high-fidelity prototypes to visualize and test concepts. Next, testing and evaluation occur through formative and summative evaluations, including usability testing and user acceptance testing to ensure the system meets requirements. Deployment involves implementation, integration, training, and launching the system. Post-deployment evaluation includes monitoring, maintenance, gathering user feedback, and implementing updates and patches based on feedback and issues, as well as planning for new releases and the system’s end-of-life [4]. Frequently, system designers employ agile methods in the software design process, which emphasizes iterative development, frequent collaboration with stakeholders, and adaptability to change throughout the project lifecycle [5].

In the context of Decision Support Systems (DSS), the contribution of HF is significant. These systems often involve complex user interfaces that must present information in a clear and actionable manner. Human-centered methodologies have advanced DSS for healthcare, aiding clinicians in making better diagnostic and therapeutic decisions and promoting patient safety [6, 7]. Yet, challenges remain in the adoption of DSS in clinical environments due to issues rooted in usability and integration into existing workflows domains where HF provides essential insights [8–11]. Absent a strong emphasis on human factors principles such as user interface design and interaction paradigms, users may not adopt these systems.

The intersection of HF and HCI is particularly potent in public health, where decisions affect large populations. Public health officials undertake complex tasks that require synthesizing vast arrays of data, and here, the role of HF is to ensure that DSS are not only functionally aligned with these tasks but are also accessible and engaging for the users. As such, DSS designed for public health need to accommodate broader determinants of health, from socioeconomic factors to healthcare services. This scoping review explores the applications of human factors in the design of evidence-based DSS in population health.

## Methods

Our scoping review was based on the methodological framework described by Arksey and O’Malley [12], with refinements by Levac and colleagues [13]. We also followed the Preferred Reporting Items for Systematic Review and Meta-Analysis Protocols (PRISMA-P and PRISMA-S, respectively) reporting guidelines to facilitate understanding and transparency [14, 15]. Our detailed study protocol was published in BMJ Open in March 2022 [16]; we briefly describe these methods below.

### Search strategy

Our search included peer-reviewed literature databases, manual searches, and grey literature. First, we searched 7 interdisciplinary indexed databases: Ovid MEDLINE, EMBASE, Scopus, PsycINFO, Compendex, IEEE Xplore, and Inspec. Our team included a librarian specialising in health science, and we further consulted with an engineering & computer science librarian to ensure both disciplines were captured. Details on our search strategy for each database can be found in the published protocol [16] and Supplemental Material. The MEDLINE search strategy was validated against a key set of 8 articles [17–24], pre-determined by the authors and was peer reviewed using PRESS [25] by another librarian, not associated with this study to ensure accuracy and comprehensiveness. We then manually searched the reference lists of included articles and relevant reviews. The search was completed in September 2023.

Our grey literature search started with a pilot review of several public health dashboards for infectious disease surveillance, modeling and forecasting, where we identified that the information presented on these websites were insufficient for the HF aspect of this review. Thus, our grey literature search included full-text conference proceedings papers, identified through Compendex (Engineering Village), IEEE Xplore, and Inspec (Engineering Village).

### Eligibility criteria

We sought to describe the HF applications to the field of population health, thus we excluded clinical applications, such as those discussing patient safety, monitoring of an individual’s health, or clinical DSS. Since HF applications in healthcare began to emerge in the 1990’s [26, 27], our search started in 1990 to capture the potential evolution of HF applications in the public health domain. As detailed in our study protocol [16], we included studies published in English since 1990 that described the development, validation, or application guided by HF principles in the field of population health. Exclusion criteria included articles whose end-user was not public health, articles not related to HF, articles that did not describe a digital evidenced-based DSS, as well as conference abstracts, reviews (including commentaries and discussion pieces), and articles not written in English.

### Screening process

The search results were integrated into Covidence [28], a systematic review management software, and duplicates were removed. Two reviewers independently screened the title and abstract of all articles according to the inclusion and exclusion criteria. Disagreements were resolved through team discussion and included a third independent reviewer as necessary. Using a similar process, selected articles then underwent full text screening by two independent reviewers, resulting in the final studies for inclusion [16].

### Data abstraction and synthesis

As outlined in the published protocol [16], a data abstraction form was developed and pilot-tested by two researchers working independently of each other. The abstracted data were synthesized according to three themes: study characteristics, population health characteristics, and human factors characteristics (Table 1). A reviewer used the form to extract data from each article; a second reviewer verified the extraction.

**Table 1:** Data abstraction themes and items

Study characteristics
1. Academic discipline of authors
a. Public health
b. Computer Science (CS)/HCI/HF/Informatics
c. GIS/Geographical Sciences
d. Multidisciplinary (combination of public health and CS/HCI/HF/Informatics or GIS/Geographical Sciences)
**2. Year of publication**
a. 2000–2005
b. 2006–2010
c. 2011–2015
d. 2016–2020
e. 2021–2023
**3. Type of publication**
a. Peer-reviewed article
b. Conference Proceeding
c. Other
**4. Publication venue**
a. Public health
b. Computer Science (CS)
c. HCI/HF
d. Informatics
e. GIS/Geographical Sciences
f. Other
**5. Study location**
a. North America
b. Central America
c. South America
d. Europe, Asia
e. Africa
f. Oceania
g. Global
**Population Health Characteristics**
**1. Population health topic area**
a. Infectious disease
b. Non-communicable disease
c. Public health data and indicators
d. Maternal, newborn, child, and family health
e. Vaccines and drugs
f. Injury
g. Mental health and substance abuse
h. Nutrition
i. Other
**2. Tool type**
a. Health surveillance
b. Program evaluation
c. Predictive modeling
d. Other
**3. Population health end-user**
a. Program planners
b. Policy makers
c. Epidemiologists
d. Community health workers
e. Academia
f. Government
g. Public health professionals not otherwise specified (NOS)
h. Multidisciplinary roles (multiple intended user groups)
i. Health care practitioners
j. Other
**4. Setting**
a. Local public health
b. Regional public health
c. Federal public health
d. Multiple levels of public health
e. Community health organizations
f. Health care organizations/hospitals
g. Other
**Human Factors Characteristics**
**1. Point in design lifecycle HF methods used**
a. Requirements gathering and analysis
b. Design and prototyping
c. Testing and evaluation
d. Post-deployment evaluation
**2. Sample size (number of participants)**
**3. Human factors study methods**
a. Questionnaires
b. Interviews
c. Focus-groups
d. Delphi discussions
e. A/B testing
f. Experiments
g. Usability testing
h. Heuristic evaluations
i. Task analysis
j. Log data for user interactions
k. Observations
l. Workshops
m. Informal feedback
n. Other
**4. Direct performance measures collected in studies using A/B testing**,** Experiments**,** and User Testing**
i. Task completion time
ii. Accuracy/Task success
iii. Efficiency
iv. Number of clicks
v. Other log data measures
vi. Mental workload

We computed descriptive statistics for all extracted items, calculating the total number and percent of all studies in a particular category. We also conducted a narrative synthesis of the included studies and the application of HF in population health.

## Results

**Fig. 1:**
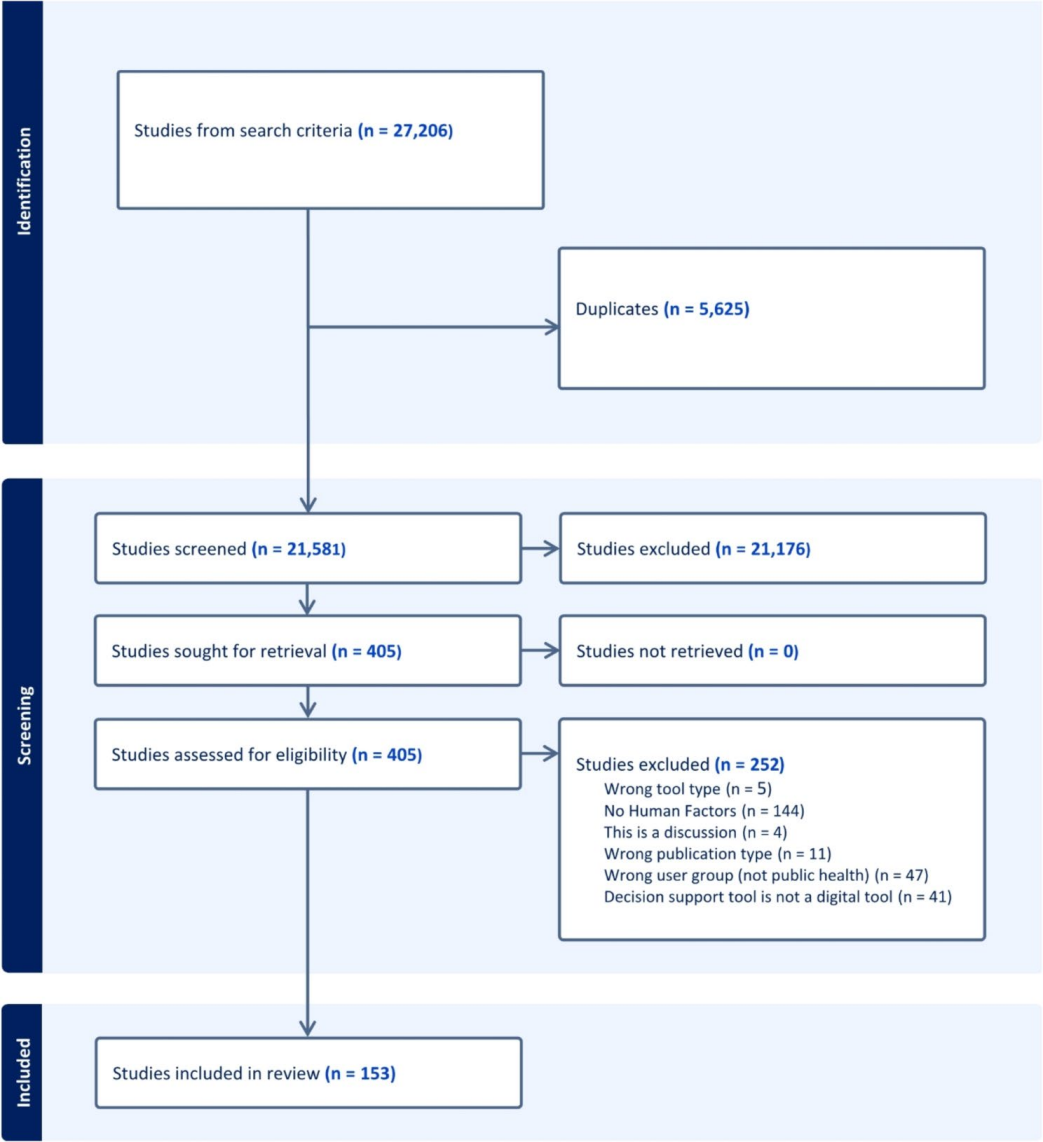
PRIMSA flow chart for screening workflow

Our search yielded 21,581 unique studies, of which 153 studies met our inclusion criteria [19, 21–24, 29–181]. Figure 1 provides a modified PRISMA flow diagram of our screening workflow. Raw data from the extraction process for the 153 included studies can be found in the Supplementary Materials.

### Study characteristics

#### Academic Discipline of authors and Year of Publication

The academic disciplines of the authors were diverse, with the majority being from Public Health (56%). Other disciplines included Multidisciplinary teams (23%), which consisted of researchers from both Public Health and Computer Science/Human-Computer Interaction/Informatics fields. Authors from solely Computer Science/Human-Computer Interaction/Human Factors or Informatics (CS/HCI/HF/Informatics) made up 20%, and those from Geographic Information Science/Geographic Science (GIS/Geographic Science) comprised 1%. The distribution of publications over the years showed that 3% were published between 2000 and 2004, 11% between 2005 and 2009, 24% between 2010 and 2014, 29% between 2015 and 2019, and 32% between 2020 and 2023 (Table 2).

#### Publication type

The types of publications varied, with peer-reviewed journal articles being the most common (76%). Conference proceedings accounted for 18% of the publications, while other types of publications made up 6% (see Table 2).

#### Publication venue type

Publications were most frequently found in Public Health (65%) venues. Additional publication venues included Informatics (13%), Computer Science/Engineering (12%), Geospatial (5%), Human Factors/Human-Computer Interaction (HF/HCI) (1%), and other disciplines (10%); see Table 2).

#### Study location

Most studies were conducted in North America (50%). Other study locations included Europe (16%), Africa (11%), Asia (10%), South America (4%), Oceania (4%), and global or multiple locations (4%; see Table 2).

**Table 2:**
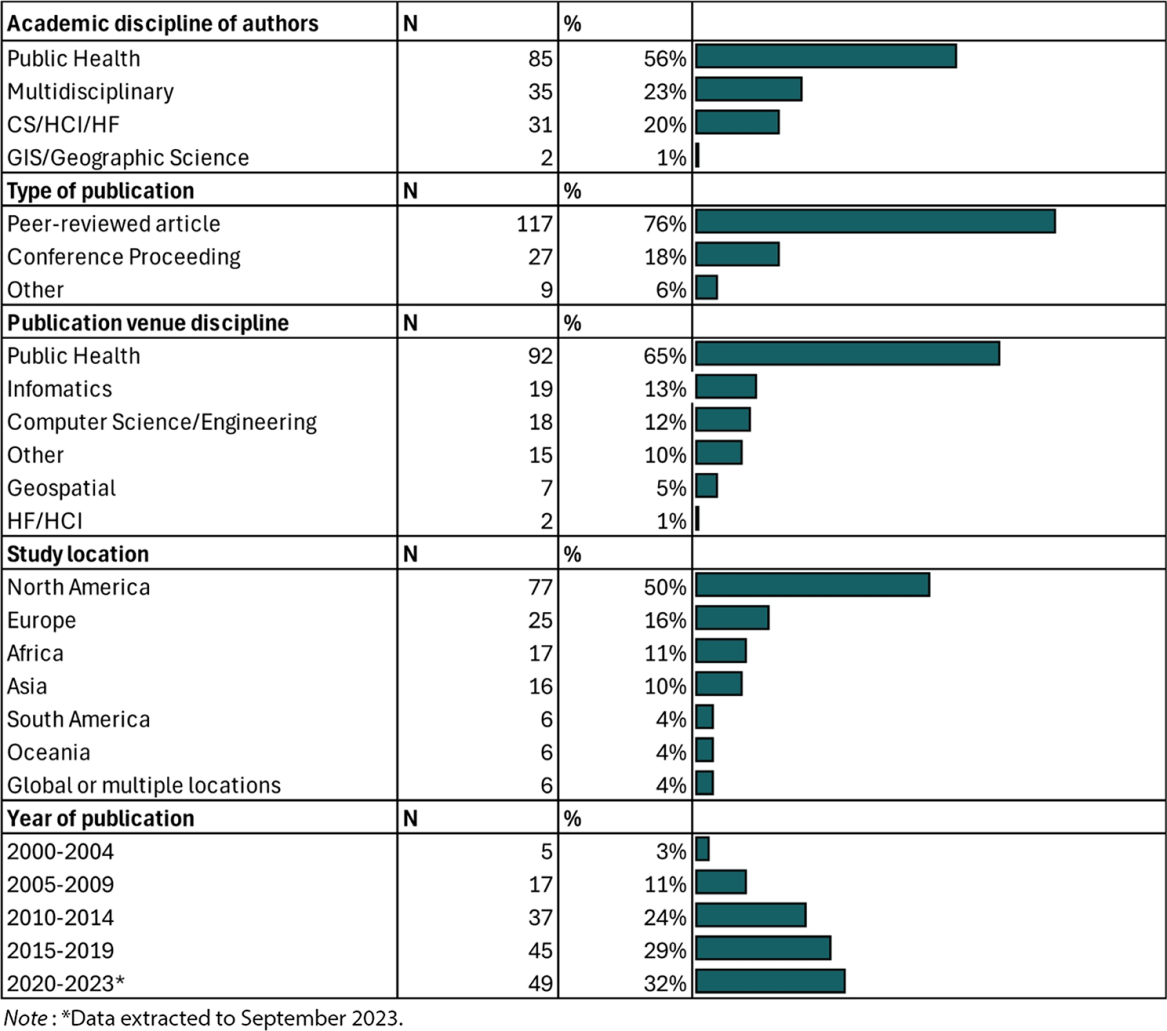
Study characteristics

### Population health characteristics

#### Population health topic area

The studies covered a range of population health topic areas. The most frequently addressed topic was infectious disease, representing 35% of the studies. Public health data and indicators were covered in 14% of studies, while maternal, newborn, and child health were the focus of 10%. Non-communicable diseases were addressed in 10% of the studies, and vaccines and drugs were the topic of 6%. Other areas included injury (4%), mental health (3%), nutrition (3%), and substance abuse (2%). Various other topics were covered in 13% of studies (Table 3).

#### DSS type

Most of the studies utilized health surveillance tools, accounting for 69% of studies. Program evaluation tools and predictive modeling tools were each used in 8% of the studies. Other types of tools were employed in 14% of the studies (see Table 3).

#### Population health end-user

The end-users of the population health tools and interventions were predominantly multidisciplinary teams, representing 35% of the studies. Program planners were the end-users in 27% of studies, while public health professionals (not otherwise specified) accounted for 12%. Policy makers were the end-users in 8% of the studies, community health workers in 4%, and academia in 3%. Other end-users were identified in 12% of studies (see Table 3).

#### Population health setting

The settings refer to where the tools are intended to be used. Multiple levels of public health were the most common setting, reported in 25% of the studies. Local public health units were the intended setting in 17% of the studies, and regional public health in 16%. Public health (not otherwise specified) was the setting in 16% of the studies, while federal public health accounted for 12%. Hospitals were the intended setting in 4% of the studies, community health centres in 3%, and other settings in 8% of studies (see Table 3).

**Table 3:**
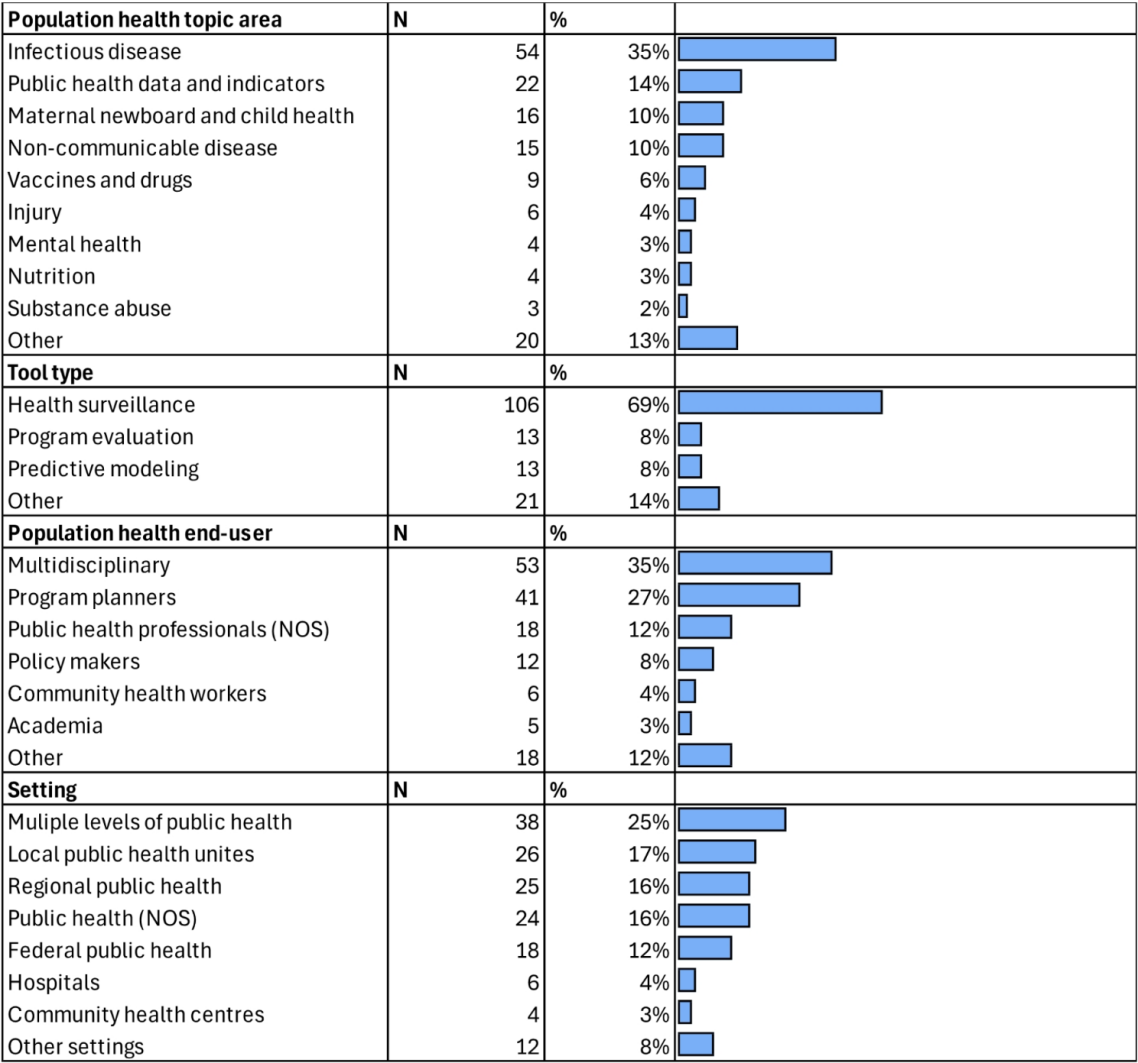
Population health study characteristics

### Human factors characteristics

Researchers primarily engaged with users during the testing and evaluation phase, followed by the post-deployment evaluation phase, the needs assessment and requirements gathering phase, and the design and prototyping phase (Table 4). The majority of studies (*n* = 96) involved users at only one point in the design lifecycle; 36 studies engaged users in two phases, 17 studies in three phases, and only 4 studies involved users in all four design lifecycle phases. Detailed results for how users were engaged within each phase are presented in the subsequent sections.

#### User needs assessment and requirements gathering

During the needs assessment and requirements gathering phase, various methods were employed to engage users and gather necessary information. Interviews were the most frequently used method, cited in 26 studies, with an average sample size of 15 participants, although 19% of these studies did not specify the sample size. Meetings, workshops, and discussions were used in 21 studies, with an average of 13 participants, but a significant number of these studies (67%) did not report sample sizes. Focus groups were conducted in 11 studies, averaging 21 participants, with 36% not specifying sample sizes. Questionnaires were used in 6 studies, with a mean sample size of 27 and all studies reporting their sample sizes. Observations and the Delphi method were each employed in 5 studies. Observations averaged at 10 participants with 20% not reporting sample sizes, while the Delphi method had a notably higher average of 84 participants, with 60% not specifying sample sizes. Less frequently used methods included usability assessments of baseline tools and task analysis (see Table 4).

#### Design and prototyping

In the design and prototyping phase, several methods were utilized to engage users and gather feedback. The most frequently used method was design-based workshops, reported in 16 studies, with an average sample size of 25 participants. Expert and stakeholder reviews were conducted in 9 studies, averaging at 3 participants. Heuristic evaluations were used in 4 studies, with an average of 4 participants. Focus groups and questionnaires were each employed in 3 studies, with focus groups averaging at 7 participants and questionnaires at 13 participants. Interviews were conducted in 2 studies with the average sample size of 18. Informal feedback was gathered in 2 studies, with an average sample size of 5 participants. The Delphi method was used in 1 study, but no information on sample size was provided. Overall, many of the qualitative methods for engaging users in the design and prototyping processes neglected to indicate their sample size (see Table 4).

#### User testing and evaluation

In the user testing and system evaluation phase, various methods and measures were employed to assess system performance and user experience. User testing, was the most frequently used method, appearing in 49 studies with an average sample size of 16 participants. Of the 49 studies that conducted user testing, 1 study [69] used an experimental framework, 11 collected quantitative data [21, 47, 48, 65, 69, 117, 132, 138, 146, 162, 177] including: task completion time (8 studies; [21, 47, 48, 65, 117, 138, 146, 177] ), task success/accuracy (6 studies; [21, 48, 65, 69, 132, 162]), efficiency (1 study; [69]), and the number of clicks (1 study; [146] ). Questionnaires were utilized in 43 studies, with an average sample size of 22 participants, while interviews were conducted in 21 studies, averaging at 14 participants. Informal feedback was gathered in 17 studies, with an average sample size of 8 participants, and focus groups were used in 12 studies, with an average of 13 participants. Log data was analyzed in 3 studies, and experiment [69] 1 study, with an average sample size of 33 participants. The Delphi method was used in 2 studies, with an average sample size of 15 participants. Notably, many studies using these methods neglected to specify their sample sizes, particularly for qualitative methods such as informal feedback sessions, like the qualitative methods applied in the design and prototyping phase (see Table 4).

#### Post-deployment evaluation

In the post-deployment assessment and evaluation phase, various methods were employed to gather feedback and assess system performance after it was deployed for use by end-users. Questionnaires were the most frequently used method, reported in 33 studies with an average sample size of 71 participants. Interviews were conducted in 28 studies, averaging at 44 participants, and focus groups were used in 9 studies with an average sample size of 22 participants. User testing was employed in 7 studies, with an average sample size of 11. Quantitative metrics were used in 4 of the 7 studies that conducted user testing and included task success/accuracy (3 studies; [23, 149, 152]), the number of clicks (1 studies; [146]), and task completion time (1 study; [146]). Additional methods included log data analysis (5 studies), informal feedback (4 studies), and observations (3 studies) with an average sample size of 15 participants. App issue reporting and experiment and A/B testing were each conducted in 2 studies, with the latter having an average sample size of 105 participants. Heuristic evaluations were used in 1 study, with an average sample size of 4 participants. Notably, again many studies, particularly those relying on qualitative methods such as informal feedback, did not specify their sample sizes (see Table 4).

**Table 4:**
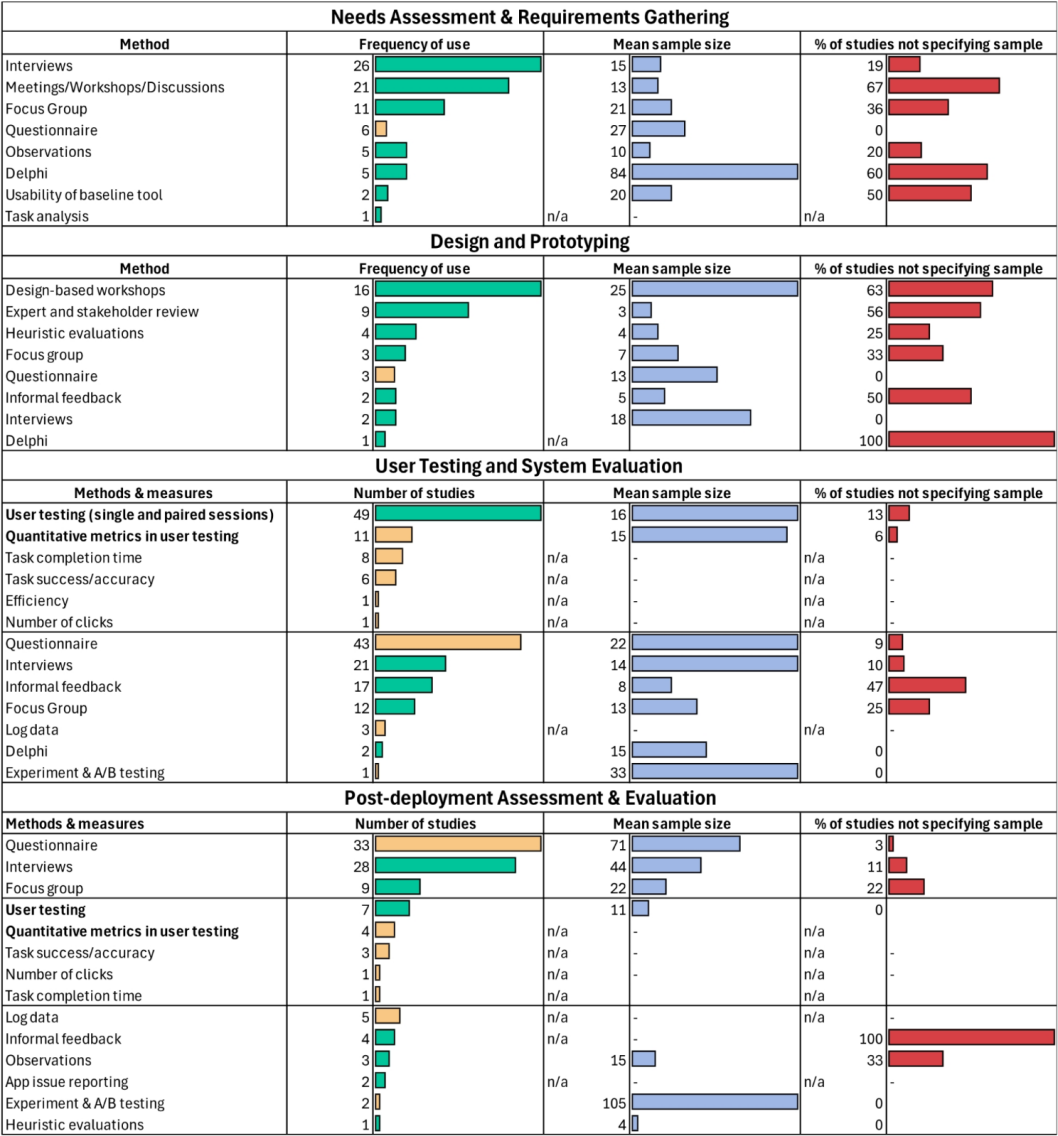
HF study characteristics. *Note* that orange bars reflect quantitative methods while green bars represent qualitative methods

## Discussion

### Have HF methods been used to their full potential?

Over the past 20 years, HF methods have been increasingly applied throughout the design lifecycle of DSS for public health contexts. A variety of qualitative and quantitative methods were used, with qualitative methods used more frequently during the needs assessment and design and prototyping phases, while quantitative methods more frequently used in the two evaluation phases: user testing and evaluation and post-deployment evaluation. Indeed, qualitative methods, such as interviews and observations, provide deep, contextual insights into user needs and behaviors, ensuring a user-centered design process. They allow for flexibility and iteration, uncovering unmet needs and fostering empathy, which leads to more inclusive and effective solutions. These methods help designers create systems that truly resonate with and benefit users, which is why they are advantageous in the early phases of the design lifecycle. On the other hand, quantitative methods provide objective, measurable data that allow for statistical analysis, and benchmarking, ensuring rigorous evaluation during user testing and post-deployment phases. These methods offer precision, reproducibility, and the ability to identify trends, enabling data-driven decisions and continuous improvement of system performance.

However, our findings indicate that researchers have not been using quantitative human factors methods to their full potential in the two evaluation phases. Importantly, most user testing and evaluation approaches did not collect direct measures of performance with the system. Additionally, only 3 studies [69, 121, 127] employed A/B testing or experimental methods to compare new or current tools with alternatives in public health contexts. Furthermore, no study evaluated whether these DSS help public health professionals make better decisions. As such, despite following some best practices in engaging users in the system design process, there is little evidence for the efficacy of these tools in supporting users in decision making tasks. Furthermore, a large proportion of studies did not report their sample size, particularly for qualitative methods. Those that reported sample sizes for qualitative studies generally followed best practices (e.g. 6–8 participants per focus group) [182]. Most studies reported sample sizes for quantitative methods, which followed best practices using larger sample sizes than qualitative methods (e.g., 20 + per questionnaire).

#### Human factors vs. agile Software Development

In the field of HF, researchers have thoroughly and rigorously assessed system design in the context of safety-critical systems such as those encountered in the aviation, surface transportation, military, and nuclear domains. However, as demonstrated in this study, this approach is lacking in the design of DSS in public health. This may in part be attributed to several constraints such as time and resources. For instance, funding opportunities are more limited for public health DSS than in other domains such as military DSS. In turn, this limits the number of public health staff available to develop and systematically evaluate these systems. Against these constraints, agile approaches to development afford user engagement and feedback throughout the design lifecycle, however, they may fall short in providing robust evidence for the efficacy of DSS. Indeed, most studies identified in this review were from researchers in the public health domain. Multidisciplinary teams may open-up additional funding opportunities in addition to fostering synergy between public health domain expertise and engineering technical skills.

#### When should we conduct HF experiments?

In systems design, it is best practice to engage with users throughout the design lifecycle. Encouragingly, we have seen an increase in the engagement of users in the design of DSS for public health. While it may not always be feasible to conduct A/B testing or experimentation, especially under time and funding constraints, some circumstances may warrant a more thorough approach. For example, more rigorous testing may be beneficial in the context of DSS intended to support high-stakes decision-making processes. Additionally, introducing novel technologies, such as artificial intelligence (AI) and machine learning (ML), in public health necessitates thorough testing to validate their efficacy. AI and ML models can potentially enhance the speed and accuracy of epidemiological insights, enabling quicker decision-making during time-critical events like the COVID-19 pandemic [183]. However, HF challenges such as the “black box” that characterises many AI/ML tools can hinder the ability of epidemiologists to explain results and decision-makers to take confident action.

### Strengths and limitations

Our scoping review has numerous strengths. Since it was designed to capture studies in both engineering and public health over the last twenty years, it has considerable breadth and comprehensiveness. Importantly, the long review period allowed us to track changes in this area over time. Our search strategy was reviewed by two librarians in both the public health sciences and engineering domains, which also improves the rigour of our search and address challenges with different nomenclature with this interdisciplinary research. We were also able to ensure that each record was reviewed by both a team member from HF engineering and one from public health, with the ability to discuss potential conflicts with a third member of the study team. This approach reduces the likelihood of false positives or negatives in terms of the studies deemed to meet inclusion criteria. Finally, our study protocol was previously peer-reviewed and published [16] and we did not deviate from our study protocol.

Our review also has some important limitations. We were only able to include studies published in English and thus we may be under-capturing studies from the Global South. Our review also does not include a full appraisal of methodological quality or risk of bias as such checklists do not exist for the study of HF in health. As DSS continue to be developed for use in clinical medicine and population health, the development of a checklist to guide rigorous Human Factors evaluations may represent a fruitful area of future work, especially by groups such as the EQUATOR network [184]. While it was difficult to summarize all potentially relevant details of our included studies due to space restrictions, we aimed to cover the most salient details for stakeholders in this space. We also present our full table of the 153 studies that met our inclusion criteria in the Supplementary Materials.

## Conclusion

While we identified many studies that applied HF methods to design decision support tools for population health, few leveraged HF methods to their full potential. We offered several recommendations for how HF methods can be leveraged at different points within the design lifecycle. The key is to engage with users early on and throughout the design process rather than simply bringing in end-users for usability testing. In terms of testing, there is a need to consider additional metrics beyond usability and tool utility. This includes measuring task performance, mental workload, situation awareness, and, ultimately, the quality of decisions made. Furthermore, there is a greater need for more rigorous evaluations, to generate the level of evidence needed to determine if and how DSS improve public health decision-making. Overall, HF methods have great potential for enhancing the impact of dashboards and data-based decision support tools and efforts are needed to adopt best practices in design and evaluation.

## Electronic supplementary material

Below is the link to the electronic supplementary material.

Supplementary Material 1

Supplementary Material 2

## Data Availability

All data generated or analyzed during this study are included in this published article and its supplementary information.

## Abbreviations

AI: Artificial Intelligence
CS: Computer Science
DSS: Decision Support Systems
HCI: Human Computer Interaction
HF: Human Factors
ML: Machine Learning
