# Supplementary material for: Human factors methods in the design of digital decision support systems for population health: a scoping review
Source: BMC Public Health. 2024 Sep 10;24:2458. doi: 10.1186/s12889-024-19968-8 (PMC11385511; doi:10.1186/s12889-024-19968-8)
Supplement: Supplementary file 1 — Supplementary Material 1 [file 12889_2024_19968_MOESM1_ESM.pdf]

Supplement 1: Search strategy

OVID MEDLINE

Database: Ovid MEDLINE: Epub Ahead of Print, In-Process & Other Non-Indexed Citations, Ovid **MEDLINE® Daily and Ovid MEDLINE® <1946**-Present> Search Strategy:

----

1 Ergonomics/ (11682)

2 exp Data Display/ (44959)

3 Man-Machine Systems/ (2819)

4 human engineering/ (11682)

5 exp user-computer interface/ (37928)

6 Universal Design/ (18)

7 Interviews as Topic/ (64486)

8 Focus Groups/ (31437)

9 (think adj aloud?).ti,ab,kf. (1092)

10 concurrent verbal protocol?.ti,ab,kf. (17)

11 ergonomic*.ti,ab,kf. (10831)

12 universal design*.ti,ab,kf. (455)

13 human performance model*.ti,ab,kf. (35)

14 cognitive task* analy*.ti,ab,kf. (196)

15 (human* adj2 (factor* or engineering)).ti,ab,kf. (22796)

16 (human* adj center* adj2 (design? or interface? or experience?)).ti,ab,kf. (272)

17 (human* adj centre* adj2 (design? or interface? or experience?)).ti,ab,kf. (68)

18 (user* adj center* adj2 (design? or interface? or experience?)).ti,ab,kf. (871)

19 (user* adj centr* adj2 (design? or interface? or experience?)).ti,ab,kf. (269)

20 ((user* or interface*) adj3 (friendly or intuit* or appeal* or informat* or understand* or need*)).ti,ab,kf. (24645)

21 ((user* or usability or stakeholder* or personnel or leader*) adj4 (eval* or experience* or involve* or test* or interview* or consult* or needs or feedback or meeting* or analy*)).ti,ab,kf. (51123)

22 ((tool* or dashboard* or interface* or prototype* or pilot*) adj4 (eval* or test* or need* or consult* or involve* or feedback* or model*)).ti,ab,kf. (98473)

23 ((cognitive or organizational or visual) adj2 ergonomic*).ti,ab,kf. (196)

24 (time adj series adj2 analy*).ti,ab,kf. (8829)

25 or/1-24 [Human Factors concept] (368120)

26 1 or 2 or 3 or 4 or 5 or 6 or 9 or 10 or 11 or 12 or 13 or 14 or 15 or 16 or 17 or 18 or 19 or 20 or 21 or 22 or 23 or 24 [Human Factors Concept without Interviews or Focus Groups] (282541)

27 Medical Informatics/ (12204)

28 Health Information Exchange/ (934)
29 exp Medical Informatics Applications/ (445167)
30 Health Information Interoperability/ (188)
31 Information Systems/ (19000)
32 Health Information Systems/ (1372)
33 Geographic Information Systems/ (8322)
34 Integrated Advanced Information Management Systems/ (289)
35 exp Medical Informatics Computing/ (1438)
36 Big Data/ (1322)
37 Community Networks/ (7026)
38 exp Decision Support Techniques/ (78829)
39 Dental Informatics/ (167)
40 Nursing Informatics/ (1534)
41 Public Health Informatics/ (1177)
42 Informatics/ (1029)
43 Consumer Health Informatics/ (118)
44 exp Data Display/ (44959)
45 data visualization/ (240)
46 exp Decision Theory/ (12359)
47 Decision Making/ (97733)
48 Decision Making, Organizational/ (11173)
49 (decision adj2 (tool or tools or support* or aid or aids or model* or analy* or mak* or theor* or system* or process* or activit* or software*)).ti,ab,kf. (198139)
50 ((information or data or visual* or audit or feedback) adj2 (display* or interface* or support* or system* or tool? or analy*)).ti,ab,kf. (524578)
51 (((real adj2 time) or audit) adj3 feedback).ti,ab,kf. (3903)
52 (indicator* or visualization* or visualisation*).ti,ab,kf. (394132)
53 informatics.ti,ab,kf. (15643)
54 dashboard?.ti,ab,kf. (1514)
55 (predicti* adj2 model*).ti,ab,kf. (69684)
56 knowledge translation.ti,ab,kf. (3407)
57 or/27-56 [Decision Support Tool concept] (1623417)
58 Global Health/ (49512)
59 exp Population Health/ (39375)
60 Public Health/ (84274)
61 exp Public Health Practice/ (720228)
62 exp Disease Outbreaks/ (144607)

63 exp Disease Transmission, Infectious/ (72994)
64 Community Health Planning/ (5162)
65 Health Status Indicators/ (23701)
66 Chronic Disease Indicators/ (18)
67 Global Burden of Disease/ (868)
68 Preventive Health Services/ (13819)
69 exp Epidemiology/ (27240)
70 "Quality of Health Care"/ (74260)
71 Quality Indicators, Health Care/ (16164)
72 Outcome Assessment, Health Care/ (75233)
73 "Outcome and Process Assessment, Health Care"/ (27745)
74 Process Assessment, Health Care/ (4831)
75 ((population or community or public) adj2 (health or medicine or surveillance or outbreak* or transmission*)).ti,ab,kf. (351133)
76 (health adj2 (department? or agency or agencies)).ti,ab,kf. (33527)
77 or/58-76 [Population Health Concept] (1517418)
78 25 and 57 and 77 [Combining all three concepts] (11801)
79 26 and 57 and 77 [Combining all three concepts minus Interviews and Focus Groups] (8039)
80 limit 78 to yr="1990 -Current" (11668)
81 limit 79 to yr="1990 -Current" [Minus Interviews and Focus Groups] (7919)
82 (Animals/ or Models, Animals/ or Disease Models, Animal/) not Humans/ (4763193)
83 ((animal or animals or veterinary* or dog or dogs or feline) not human*).ti,ab,kf. (1030290)
84 82 or 83 (5038448)
85 80 not 84 [exclude animal studies] (11443)
86 79 not 84 [exclude animal studies and minus Interviews and Focus Groups] (7828)
87 exp Meta-Analysis as Topic/ (21211)
88 Meta-Analysis/ (127629)
89 Review Literature as Topic/ (7892)
90 Systematic Review/ (147108)
91 Systematic Reviews as Topic/ (4829)
92 (meta analy$ or metaanaly$ or systematic review$ or systematic overview$ or scoping review$ or umbrella review$).ti,ab,kf. (312668)
93 (cochrane or embase or psychlit or psyclit or psychinfo of psycinfo or cinahl or cinhal or science citation index or scopus or web of science or bids or cancerlit or ageline).ab. (167161)
94 (reference list$ or bibliograph$ or hand-search$ or relevant journal$ or manual search$).ab. (46199)

95 (review or editorial or guideline or letter or meta analysis or news* or patient education handout).pt. (4736370)

96 or/87-95 [Modified SIGN filter to retrieve systematic reviews; modified to expand to other reviews as well as non-journal articles] (4858052)

97 85 and 96 [set of reviews and non-primary literature for team to review for background information] (1184)

98 86 and 96 [set of reviews and non-primary literature for team to review for background information minus Interviews and Focus Groups] (1094)

99 85 not 96 [Remove secondary studies and non-empirical studies] (10259)

100 86 not 96 [Remove secondary studies and non-empirical studies minus Interviews and Focus Groups] (6734)

101 99 not 100 [Team to look through articles not included by not including Interviews and Focus Groups - to see if there are important or studies here that should be found/retrieved] (3637)

OVID EMBASE

Database: Embase Classic+Embase <1947 to 2021 March 09>

Search Strategy:

----

1 ergonomics/ (12263)
2 human machine interface/ (164)
3 man machine interaction/ (3583)
4 human computer interaction/ (6331)
5 universal design/ (174)
6 time series analysis/ (28615)
7 (think adj aloud?).ti,ab,kw. (1326)
8 concurrent verbal protocol?.ti,ab,kw. (21)
9 ergonomic*.ti,ab,kw. (16403)
10 universal design*.ti,ab,kw. (552)
11 human performance model*.ti,ab,kw. (49)
12 cognitive task* analy*.ti,ab,kw. (269)
13 (human* adj2 (factor* or engineering)).ti,ab,kw. (28921)
14 (human* adj center* adj2 (design? or interface? or experience?)).ti,ab,kw. (333)
15 (human* adj centre* adj2 (design? or interface? or experience?)).ti,ab,kw. (100)
16 (user* adj center* adj2 (design? or interface? or experience?)).ti,ab,kw. (889)
17 (user* adj centr* adj2 (design? or interface? or experience?)).ti,ab,kw. (365)
18 ((user* or interface*) adj3 (friendly or intuit* or appeal* or informat* or understand* or need*)).ti,ab,kw. (30736)
19 ((user* or usability or stakeholder* or personnel or leader*) adj4 (eval* or experience* or involve* or test* or interview* or consult* or needs or feedback or meeting* or analy*)).ti,ab,kw. (68410)
20 ((tool* or dashboard* or interface* or prototype* or pilot*) adj4 (eval* or test* or need* or consult* or involve* or feedback* or model*)).ti,ab,kw. (137959)
21 ((cognitive or organizational or visual) adj2 ergonomic*).ti,ab,kw. (275)
22 (time adj series adj2 analy*).ti,ab,kw. (10697)
23 or/1-22 [Human Factors Concept] (315579)
24 decision making/ (245242)
25 decision support system/ (23523)
26 decision theory/ (1803)
27 "decision tree"/ (14435)
28 multicriteria decision analysis/ (378)
29 computer interface/ (32621)

30 data visualization/ (992)
31 visual display unit/ (1285)
32 expert system/ (5487)
33 information system/ (39837)
34 medical informatics/ (21188)
35 medical information system/ (21564)
36 feedback system/ (87793)
37 (decision adj2 (tool or tools or support* or aid or aids or model* or analy* or mak* or theor* or system* or process* or activit* or software*)).ti,ab,kw. (274740)
38 ((information or data or visual* or audit or feedback) adj2 (display* or interface* or support* or system* or tool? or analy*)).ti,ab,kw. (758501)
39 (((real adj2 time) or audit) adj3 feedback).ti,ab,kw. (5687)
40 (indicator* or visualization* or visualisation*).ti,ab,kw. (530927)
41 informatics.ti,ab,kw. (20484)
42 dashboard?.ti,ab,kw. (2860)
43 (predicti* adj2 model*).ti,ab,kw. (94976)
44 knowledge translation.ti,ab,kw. (4632)
45 or/24-44 [Decision Support Concept] (1909062)
46 global health/ (12046)
47 population health/ (3460)
48 population health management/ (192)
49 public health/ (203195)
50 public health service/ (76473)
51 public health systems research/ (93)
52 public health problem/ (16002)
53 global disease burden/ (3341)
54 health care planning/ (104835)
55 exp health promotion/ (103435)
56 epidemiology/ (235679)
57 exp disease surveillance/ (31920)
58 exp mass screening/ (261124)
59 preventive medicine/ (28808)
60 preventive health service/ (29836)
61 ((population or community or public or global or provincial* or national* or international* or region*) adj2 (health or medicine or surveillance or outbreak* or transmission*)).ti,ab,kw. (548142)
62 (health adj2 (department? or agency or agencies)).ti,ab,kw. (42312)

63 or/46-62 [Population Health Concept] (1380107)

64 23 and 45 and 63 [Combine all three concepts] (7385)

65 limit 64 to english language (7175)

66 limit 65 to yr="1990 -Current" (7079)

67 limit 66 to (article in press or books or chapter or conference abstract or conference paper or "conference review" or editorial or erratum or letter or note or "review" or short survey or tombstone) (2414)

68 66 not 67 (4665)

****

APA PsycINFO

Database: APA PsycInfo <1806 to March Week 1 2021>

Search Strategy:

----

1 human factors engineering/ (8871)

2 engineering psychology/ (982)

3 computer assisted design/ (545)

4 human computer interaction/ (11076)

5 human computer interaction measures/ (4)

6 human factors measures/ (46)

7 exp human machine systems/ (7810)

8 human technology interaction/ (170)

9 human computer interaction/ (11076)

10 human computer interaction measures/ (4)

11 (think adj aloud?).tw. (1991)

12 concurrent verbal protocol?.tw. (55)

13 ergonomic*.tw. (4413)

14 universal design*.tw. (593)

15 human performance model*.tw. (79)

16 cognitive task* analy*.tw. (305)

17 (human adj2 (factor* or engineer*)).tw. (6140)

18 (human* adj center* adj2 (design? or display* or interface? or experience?)).tw. (167)

19 (human* adj centre* adj2 (design? or display* or interface? or experience?)).tw. (55)

20 (user* adj center* adj2 (design? or display* or interface? or experience?)).tw. (442)

21 (user* adj centr* adj2 (design? or display* or interface? or experience?)).tw. (181)

22 ((user* or interface*) adj3 (friendly or intuit* or appeal* or informat* or understand* or need*)).tw. (8880)

23 ((user* or usability or stakeholder* or personnel or leader*) adj4 (eval* or experience* or involve* or test* or interview* or consult* or needs or feedback or meeting* or analy*)).tw. (38987)

24 ((tool* or dashboard* or interface* or prototype* or pilot*) adj4 (eval* or test* or need* or consult* or involve* or feedback* or model*)).tw. (25898)

25 ((cognitive or organizational or visual) adj2 ergonomic*).tw. (225)

26 (time adj series adj2 analy*).tw. (2613)

27 or/1-26 [Human Factors Concept] (100216)

28 exp decision making/ (124921)

29 decision support systems/ (3388)

30 exp displays/ (11387)

31 exp decision theory/ (1390)

32 information systems/ (5553)

33 (decision adj2 (tool or tools or support* or aid or aids or model* or analy* or mak* or theor* or system* or process* or activit* or software*)).tw. (120042)

34 ((information or data or visual* or audit or feedback) adj2 (display* or interface* or support* or system* or tool? or analy*)).tw. (171919)

35 (((real adj2 time) or audit) adj3 feedback).tw. (662)

36 (indicator* or visualization* or visualisation*).tw. (78016)

37 informatic*.tw. (1691)

38 dashboard*.tw. (321)

39 (predicti* adj2 model*).tw. (11571)

40 knowledge translation.tw. (935)

41 or/28-40 [Decision Support Concept] (430017)

42 exp population health/ (692)

43 exp public health/ (33986)

44 health promotion/ (25551)

45 global health/ (2471)

46 exp preventive health services/ (3433)

47 epidemiology/ (51345)

48 ((population or community or public) adj2 (health or medicine or surveillance or outbreak* or transmission*)).tw. (78437)

49 (health adj2 (department? or agency or agencies)).tw. (7870)

50 (outbreak* or epidemic* or pandemic* or chronic disease*).tw. (32171)

51 or/42-50 [Population Health Concept] (184237)

52 27 and 41 and 51 (977)

53 limit 52 to yr="1990 -Current" (968)

54 (dissertation abstract or edited book).pt. (866399)

55 53 not 54 (737)

****

Scopus

(TITLE-ABS-KEY ("global health" OR (community OR public OR population* OR prevent*) W/2 (health OR medicine OR surveillance OR outbreak* OR transmission*) OR "health W/2 (department* OR agency OR agencies OR organisation* OR organization* OR provinc* OR federal* OR government*)" OR pandemic* OR epidemic* OR epidemiolog* OR "health promot*" OR "accident* w/2 prevent*" OR "disease* w/2 outbreak*" OR "disease* w/2 transm*" OR "mass screening*")) AND (TITLE-ABS-KEY (ergonomic* OR "human factor*" OR "human w/2 (factor* or engineer*)" OR "human w/3 (design* or interface* or display* or data)" OR "user* w/3 (design* or interface* or display* or data)" OR "concurrent verbal protocol*" OR "universal design" OR "human performance model*" OR "cogniti* task* analy*" OR "think w/1 aloud*" OR "(user* or interface*) adj3 (friendly or intuit* or appeal* or informat* or understand* or need* or design* or eval*)" OR "(tool* or dashboard* or interface* or prototype* or pilot* or display*) w/4 (eval* or test* or need* or consult* or involv* or feedback* or model*)" OR "time series analy*" OR "(user* or usability or stakeholder* or personnel or leader*) w/4 (eval* or experience* or involv* or test* or interview* or consult* or needs or feedback or meeting* or analy*)" OR "visual analy*" OR prototype* OR usability)) AND (TITLE-ABS-KEY (decision* OR dashboard* OR display* OR informatic* OR "data visual*" OR interface* OR "information system*" OR "predict* model*" OR "knowledge translation" OR "audit w/3 feedback" OR "(information or data or visual* or audit or feedback*) w/4 (display* or interface* or support* or system* or tool? or analy*)" OR model* OR simulation* OR visualization* OR visualisation* OR "real W/3 feedback*")

COMPENDEX

((((($Decision $Support $Systems OR $Decision $Making OR $Information $Systems OR $Visualization OR $Graphical $User $Interfaces OR $User $Interfaces OR $Interactive $Computer $Systems) WN CV)) OR (($Dashboard OR $Platform OR $Interface OR $Display) WN KY)) AND (1666-2022 WN YR)) AND ((((($Public $Health) WN CV)) OR (($Population $Health OR $Community $Health OR $Community $Medicine) WN KY)) AND (1666-2022 WN YR)) AND ((((($Human $Engineering OR $Ergonomics OR $User $Experience OR $Human $Computer $Interaction OR $Usability $Engineering) WN CV)) OR (($Human Factor* OR $Usability OR $User Friendl* OR $Cognitive Ergonomic*) WN KY)) AND (1666-2022 WN YR))

INSPEC

(((($Public $Health) WN CV)) OR (($Population $Health OR $Community $Health OR $Community $Medicine) WN KY)) AND (1896-2022 WN YR)) AND (((($Decision $Making OR $Decision $Support $Systems OR $User $Interfaces OR $Graphic $User $Interfaces OR $Interactive $Systems OR $Data $Visualization) WN CV)) OR (($Dashboard OR $Interface OR $Display OR $Decision $Support System*) WN KY)) AND (1896-2022 WN YR)) AND (((($Human $Factors OR $Ergonomics OR $Human $Computer $Interaction) WN CV)) OR (($Usability OR $User $Experience OR $User $Evaluation OR $Cognitive $Ergonomics) WN KY)) AND (1896-2022 WN YR))

IEEE Xplore

"All Metadata":Human Factors OR "All Metadata":Ergonomics OR "All Metadata":Cognitive Ergonomics OR "All Metadata":Human Computer Interaction OR "All Metadata":User Experience OR "All Metadata":Usability OR "All Metadata":User Friendly) AND ("All Metadata":Dashboard OR "All Metadata":Interface OR "All Metadata":Data Visualization OR "All Metadata":Decision Support) AND ("All Metadata":Public Health OR "All Metadata":Population Health OR "All Metadata":Community Health)
